# Clinical characteristics of anti‐neurofascin 155 antibody‐positive autoimmune nodopathy in children

**DOI:** 10.1002/ped4.70008

**Published:** 2025-04-23

**Authors:** Liya Cui, Shuai Gong, Yongxiang Zhao, Siwei Wang, Xinying Yang, Shen Zhang, Weihua Zhang, Jiuwei Li, Bingbing Jia, Xiaona Fu, Lin Ge, Junlan Lv, Yun Peng, Hui Xiong

**Affiliations:** ^1^ Department of Neurology Beijing Children's Hospital, Capital Medical University, National Center for Children's Health Beijing China; ^2^ Department of Radiology Beijing Children's Hospital, Capital Medical University, National Center for Children's Health Beijing China; ^3^ Department of Ultrasound Beijing Children's Hospital, Capital Medical University, National Center for Children's Health Beijing China

**Keywords:** Autoimmune nodopathy, Anti‐neurofascin 155 antibody, Children, Chronic inflammatory demyelinating polyradiculoneuropathy, Clinical characteristics

## Abstract

**Importance:**

Anti‐neurofascin (anti‐NF) 155 antibody‐positive autoimmune nodopathy is a distinct subset of chronic inflammatory demyelinating polyradiculoneuropathy (CIDP). Given the increase in pediatric cases, understanding this condition is crucial.

**Objective:**

This study aimed to delineate the clinical features of children with anti‐NF155 antibody‐positive autoimmune nodopathy to enhance disease management strategies.

**Methods:**

We conducted a retrospective cohort study of 34 CIDP patients admitted to Beijing Children's Hospital from January 2015 to December 2024, including six with confirmed anti‐NF155‐antibody positivity. Their clinical symptoms, laboratory results, neuroimaging findings, and therapeutic responses were retrospectively analyzed.

**Results:**

Of the 34 patients, six (17.6%) were tested positive for anti‐NF155 antibodies. The cohort was male‐dominated (male‐to‐female ratio of 4:2) with symptoms starting primarily in school‐aged children. The symptoms included progressive limb weakness, sensory ataxia, and tremors. Notably, cerebrospinal fluid (CSF) protein levels were significantly elevated in seropositive patients. Electrophysiological studies indicated sensorimotor polyneuropathy, and neuroimaging revealed nerve root thickening. While intravenous immunoglobulin (IVIG) therapy was not effective, a combination of glucocorticoids, rituximab, and plasma exchange showed promise. At the final follow‐up, all patients experienced symptom relief and could perform daily activities without relapse.

**Interpretation:**

Pediatric anti‐NF155 antibody autoimmune nodopathy was uncommon, featuring male dominance, and distal weakness with sensory symptoms. Additionally, the CSF protein levels were significantly elevated in seropositive patients. As IVIG treatment was ineffective, early immunosuppressive therapy was recommended. Early diagnosis and treatment are critical in reducing myelin and axonal damage.

## Graphical abstract

Pediatric anti‐neurofascin 155 antibody autoimmune nodopathy was uncommon, featuring distal limb weakness with sensory symptoms. Cerebrospinal fluid protein levels were elevated, and neuroimaging revealed nerve root thickening. As intravenous immunoglobulin was ineffective, early immunosuppressive therapy was recommended.

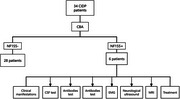

## INTRODUCTION

Chronic inflammatory demyelinating polyradiculoneuropathy (CIDP) is an acquired immune‐mediated demyelinating disorder of the peripheral nervous system. Autoimmune nodopathy involves autoantibodies, including neurofascin (NF) 155, NF186, contactin‐1 (CNTN1), and contactin‐associated protein 1 (CASPR1). The task force of the European Academy of Neurology/Peripheral Nerve Society (EAN/PNS) proposed to term “autoimmune nodopathies” to reflect their distinct clinical characteristics, which differ from the typical CIDP features such as significant inflammation or macrophage infiltration.^1^, ^2^, ^3^, ^4^ Notably, the anti‐NF155 antibody has been identified as having superior specificity and sensitivity, predominantly arising from the immunoglobulin (IgG) 4 subclass.^5^

The incidence of CIDP in children is approximately 0.22/100 000 in European populations.^6^, ^7^, ^8^ Anti‐NF155‐antibody‐positive autoimmune nodopathy presents distinct clinical symptoms, especially in younger individuals.^8^ However, a paucity of documented pediatric cases exists, particularly among Chinese patients, which has led to a gap in our understanding of the specific manifestations of the disease in young populations. To enhance our knowledge and facilitate early diagnosis and intervention, we retrospectively analyzed comprehensive data from six pediatric patients diagnosed with anti‐NF155‐positive autoimmune nodopathy.

## METHODS

### Ethical approval

The study was reviewed and approved by the Institutional Review Boards of Beijing Children's Hospital ([2024]‐E‐155‐R). Written informed consent to participate in this study was obtained from the participants’ legal guardians.

### Diagnostic criteria

All patients met the following criteria: (1) fulfillment of the 2021 EAN/PNS criteria^4^ for autoimmune nodopathy; (2) positive serum anti‐NF155 antibody; (3) negative for other Ranvier's autoantibodies; (4) under 18 years of age; (5) exclusion of patients with genetic diseases, tumors, or other autoimmune diseases.

### Participants

Thirty‐four children with CIDP were hospitalized in the Neurology Department of Beijing Children's Hospital between January 2015 and December 2024. The patients underwent comprehensive diagnostic procedures, including serum autoantibody assessments, cerebrospinal fluid (CSF) examination, neuro ultrasound, spinal cord magnetic resonance imaging (MRI), brain MRI, electromyography (EMG), and additional relevant biochemical tests.

### Therapeutic effect and follow‐up

Therapeutic responses were categorized as effective, partially effective, or ineffective. Effectiveness implied that the modified Rankin scale (mRS) score improved by at least one point. If no change was noted in the mRS score but the neurological examination results improved, we classified it as partially effective. All other responses were considered ineffective.

All patients were followed up in the outpatient clinic, with a final follow‐up in December 2024. The Inflammatory Rasch Overall Disability Scale (IRODS), Inflammatory Neuropathy Cause and Treatment (INCAT), mRS, and Medical Research Council (MRC) scales were used to perform a comprehensive assessment of all patients. The higher the scores on the aforementioned scales, the milder the condition.

### Statistical analysis

All data were statistically analyzed using IBM SPSS Statistics for Windows version 26.0. Categorical variables were analyzed using the chi‐square or Fisher's exact test. Non‐normally distributed continuous data are represented as median (interquartile range [IQR]) and were analyzed statistically using the Mann‐Whitney test. Two‐sided *P*‐values were calculated for all analyses and statistical significance was set at *P* < 0.05.

## RESULTS

### Clinical manifestations

Six of the 34 (17.6%) children with CIDP met the criteria for anti‐NF155 autoimmune nodopathy, including four males and two females. Among the six children studied, five exhibited chronic‐onset symptoms, whereas one (patient 6) had subacute onset. Additionally, three children (patients 2, 4, and 6) had a history of prodromic infections prior to symptom onset. All patients presented with symmetrical, progressive muscle weakness in all four limbs, with the lower limbs being particularly affected. Notably, patient 4 was unable to walk. Furthermore, motor and sensory deficits were particularly pronounced in the distal limbs. Additionally, all six pediatric patients developed progressive sensory ataxia. The clinical presentation was complemented by additional symptoms including numbness, tremors, and hyperalgesia (Table 1).

**TABLE 1:** Clinical features of anti‐NF155 antibody‐positive autoimmune nodopathy

											Muscle strength^*^							
No.	Sex	Age^†^ (y)	Antibody titer	CSF protein (mg/L)	Last follow‐up (month)	Weakness (B/L)	Ataxia (B/L)	Numbness (B/L)	Hyperalgesia (B/L)	Tremor (B/L)	PUL (B/L)	DUL (B/L)	PLL (B/L)	DLL (B/L)	Treatment	mRS (B/L)	INCAT (B/L)	IRODS (B/L)
1	M	8	1:10	1778	12	+/−	+/−	−/−	−/−	−/−	4^−^/5	4/5	4^−^/5	4/5	g+r	3/0	2/0	8/0
2	M	13	1:10	1455	37	+/+	+/−	+/−	−/+	−/−	5/4	4/4	5^−^/4^+^	4^+^/4^+^	g+r+p	4/1	4/0	12/0
3	M	9	1:100	1529	45	+/−	+/−	−/−	−/−	+/−	5/5	5/5	4/5	4^−^/5	g+r+i	2/0	1/0	7/0
4	F	11	1:32	4347	26	+/+	+/−	+/−	−/−	−/−	4/5^−^	3/4	3/4	0/4	g+r+i+p	4/2	7/1	17/4
5	F	12	1:32	2424	18	+/−	+/−	−/−	+/−	−/−	5/5	5/5	5/5	3^+^/5	g+r+i	3/1	2/1	8/4
6	M	10	1:10	590	37	+/−	+/−	−/−	−/−	−/−	5/5	5/5	4/5	3^+^/5	g+i	3/0	1/0	7/0

Abbreviations: B/L, before treatment/last follow‐up; CSF, cerebrospinal fluid; DLL, distal lower limbs; DUL, distal upper limbs; g, glucocorticoid; i, intravenous immunoglobulin; INCAT, inflammatory neuropathy cause and treatment (total score between 0–10); IRODS, Inflammatory Rasch built Overall Disability Scale (total score between 0 and 30); M, male; F, Female; mRS, modified Rankin scale; No, patient number; p, plasma exchange; PLL, proximal lower limbs; PUL, proximal upper limbs; r, rituximab.

^*^ Symptom onset age.

^†^ Muscle strength is evaluated by Medical Research Council scales.

### CSF test

CSF analysis was performed for all 34 patients. The quantified protein levels ranged from 590 to 4347 mg/L (reference value: 20–450 mg/L). CSF protein levels and age at symptom onset differed significantly between patients with anti‐NF155 autoimmune nodopathy and CIDP (Table 2).

**TABLE 2:** Comparison of NF155^+^ and chronic inflammatory demyelinating polyradiculoneuropathy (CIDP) patients

Characteristics	NF155^+^ (*n* = 6)	Typical CIDP (*n* = 28)	*P*‐Value^†^
Age^‡^ (year)	10.5 (2.5)	7.0 (5.5)	0.014
Male/Female	4/2	16/12	1.000
CSF protein (mg/L)	1492.0 (953.0)	825.5 (834.3)	0.038

Data are presented as median (interquartile range) or *n*.

Abbreviations: CIDP, chronic inflammatory demyelinating polyradiculoneuropathy; CSF, cerebrospinal fluid; NF155^+^, anti‐neurofascin 155 antibody‐positive autoimmune nodopathy.

^†^ Mann‐Whitney *U* test for age, and CSF protein; chi‐squared test for male/female.

^‡^ Symptom onset age.

### Antibodies test

Serum and/or CSF specimens were sent to the Beijing High Trust Diagnostic Laboratory to detect nodal and paranodal antibodies (NF186, NF155, CNTN1, and CASPR1) using the cell‐based assay. Results revealed that all six patients tested positive for anti‐NF155 antibodies in their serum and two also tested positive for anti‐NF155 antibodies in the CSF.

### EMG

EMG was conducted in all six patients, revealing heterogeneous patterns of peripheral nerve damage. All the patients exhibited varying degrees of motor and sensory nerve damage. The F‐wave latency demonstrated absent or abnormally prolonged intervals. EMG studies have also captured variable degrees of conduction block and temporal dispersion among affected individuals. Collectively, the comprehensive EMG findings indicated pronounced involvement of motor and sensory functions in the lower limbs (Table 3).

**TABLE 3:** Electrophysiological study of six patients

Patient number	Median motor DL/CV	Ulnar motor (DL/CV)	Tibial motor (DL/CV)	Peroneal motor (DL/CV)	Median sensory (DL/CV)	Ulnar sensory (DL/CV)	Peroneal sensory (DL/CV)	Conductive block	Temporal dispersion	F wave latency
P1	6.3↑/N	N/44.2↓	9.3↑/N	10.3↑/N	N/48.9↓	N/43.2↓	N/N	Yes	No	31.8
P2	9.5↑/45.8↓	6.9↑/28.1↓	14.7↑/38.7↓	11.5↑/30.4↓	ND	ND	N/47.3	No	Yes	ND
P3	4.5↑/N	N/N	12.8↑/37.0↓	9.9↑/36.9↓	N/N	N/N	N/N	Yes	No	ND
P4^†^	6.4↑/23.8↓	6.0/25.5↓	ND	ND	ND	ND	ND	No	Yes	ND
P4^‡^	N/N	N/41.3↓	ND	ND	N/47.1↓	N/37.6↓	ND	No	Yes	29.3
P5	5.2↑/28.2↓	N/29.5↓	ND	12.5↑/19.1↓	ND	ND	ND	Yes	No	ND
P6	4.1↑/N	N/N	6.3↑/N	4.3↑/44.9↓	N/45.5↓	N/44.5↓	N/40.9↓	No	No	25.9

Nerve conduction parameters are from the right limbs. F wave latency parameters are from the median motor.

Abbreviations: CV, conduction velocity (m/s); DL, distal latency (ms); N, normal; ND, undetectable.

The normal values are as follows: DL < 4 ms, motor nerve CV in the upper limb > 50 m/s, motor nerve CV of the tibial nerve > 40 m/s, motor nerve CV of the peroneal nerve > 45 m/s, and sensory nerve CV > 50 m/s.

^†^ Before treatment.

^‡^ One year after treatment.

### Neurological ultrasound

Neurological ultrasound examinations were performed on four patients (patients 1–4). Intranerve cross‐sectional area variability (INV) of the limbs was elevated, with an increase of 100%–150% compared to normal values.

### Spinal cord MRI

**FIGURE 1:**
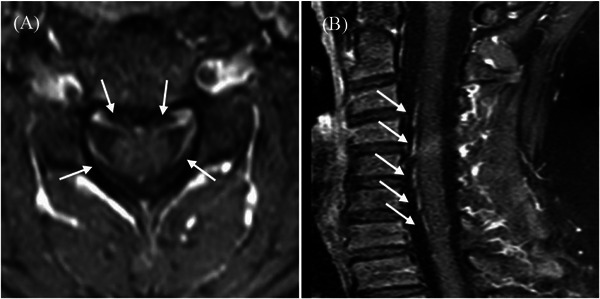
The cervical spine transverse (A) and sagittal (B) magnetic resonance imaging in patient 3 showed thickening of the spinal nerve roots with enhancement (white arrows).

All six patients underwent cervical, thoracic, and lumbar spinal cord contrast‐enhanced MRI, which revealed varying degrees of thickening of the spinal nerve roots (Figure 1).

### Diagnosis and treatment

Four patients were initially diagnosed as having CIDP. Patient 3 was initially diagnosed with Guillain‐Barré syndrome (GBS) due to its subacute onset, while patient 4 was initially diagnosed with plausible peripheral inflammatory neuropathy. All patients were treated with glucocorticoids. The mRS was used to evaluate the therapeutic response. Patient 6 was categorized as having an effective response, while patient 1 was deemed to have an ineffective response. The remaining four patients were categorized as having a partial response and subsequently received intravenous immunoglobulin (IVIG) therapy. However, after one course of IVIG treatment, reassessment showed that their conditions remained ineffective. Consequently, five patients (except patient 6) were treated with rituximab at a dose of 375 mg/m^2^ weekly. Patients 2 and 4, who had initial high mRS scores (both scoring 4) and did not respond to glucocorticoids or IVIG, received combined treatments with rituximab and plasma exchange. After four doses of treatment, the responses of these five patients were re‐evaluated and categorized as effective.

### Follow‐up

The final follow‐up was conducted in December 2024, with a duration ranging from 12 to 45 months, and a mean follow‐up duration of 29 months from the initiation of treatment. At the last follow‐up, four patients (patients 1, 2, 3, and 6) were found to be asymptomatic, and the remaining two patients experienced a significant alleviation of their clinical symptoms. All patients stopped glucocorticoid therapy, and four (patients 1, 2, 4, and 5) continued rituximab treatment. Across all patients, notable declines were observed in their mRS (average reduction of 2.5), IRODS (average reduction of 2.5), and INCAT scores (average reduction of 8.5). These findings collectively suggest the efficacy of the treatment regimen.

## DISCUSSION

Recent studies have indicated that NF155 autoantibodies may be present in pediatric patients. Among our cohort of 34 patients, we identified six seropositive patients. Within this cohort, four (66.7%) were male and two (33.3%) were female. No differences were observed in sex between patients with anti‐NF155 antibody autoimmune nodopathy and those with CIDP. Ogata et al.^9^ reported a male‐to‐female ratio of 2.1:1 among 22 Japanese patients, whereas other studies identified only male participants.^10^, ^11^ Conversely, a substantial number of researchers argue against significant gender bias.^12^, ^13^, ^14^, ^15^, ^16^ A widely accepted notion is that anti‐NF155 antibody autoimmune nodopathy typically occurs in young patients.^14^, ^17^

In the present study, we observed that five patients exhibited chronic disease onset, whereas one patient presented with subacute onset. This suggests that these two temporal onset patterns are predominant modes of disease initiation. Notably, all patients experienced symmetric, progressive weakness in the lower extremities, concurrent with sensory ataxia. Tremors, commonly reported in adult patients positive for anti‐NF155 antibodies, are typically characterized by low frequency and high amplitude.^18^, ^19^ NF155 is crucial for the formation of paranodal axoglial junctions and axonal swelling, as well as for the degeneration of cerebellar Purkinje axons.^20^ This indicates a role for NF155 in the coordination of motor function, which may be one of the causes of tremors.^11^ Some patients present with trigeminal nerve thinking, leading to facial sensory deficits.^9^, ^21^ However, only one child in our study demonstrated tremors and none of the patients presented with facial sensory deficits.

In this investigation, we observed that patients with anti‐NF155 antibody autoimmune nodopathy exhibited notably elevated protein levels in their CSF when compared to those in the classical CIDP group. Nevertheless, due to the limited number of patients involved, the sample size was insufficient to infer population‐level trends, warranting a need for further expansion of the study to draw more robust conclusions.

EMG revealed conduction impairments in both motor and sensory pathways, which are commonly observed in the lower extremities. We observed prolonged motor nerve conduction latency and slow conduction velocity, indicating peripheral nerve damage. A conduction block and temporal dispersion were also observed. Four patients failed to elicit F‐waves, indicating a certain degree of proximal nerve injury. Notably, patient 4 demonstrated significant prolongation of upper limb motor nerve latency, reduced conduction velocity, undetectable sensory nerve conduction, and F‐wave latency. One year later, the impairments had largely resolved, suggesting lesion reversibility. However, motor and sensory conduction in the lower limbs remained unelicited, suggesting a severe involvement of the lower extremities, which required additional time for recovery.

Our study also demonstrated nerve root thickening on both neuro ultrasound and spinal cord MRI. Four patients in our cohort who underwent nerve ultrasonography revealed an increase in the INV in the extremities. Previous studies have demonstrated that in adult patients with CIDP, increased INV correlates with aggravating clinical conditions, whereas stable or remitting disease courses exhibit no significant changes in INV.^22^, ^23^, ^24^, ^25^ Some researchers have suggested that variations in individual immune responses may account for differences in INV readings.^26^, ^27^ Ultrasound is distinguished by its noninvasive nature, ease of use, time efficiency, and cost‐effectiveness, making it a valuable tool for monitoring conditions and follow‐up. However, a significant limitation is the absence of internationally recognized standards for normal nerve root values across different pediatric age groups. To mitigate this issue and minimize discrepancies and potential errors, performing these evaluations within the same children's hospitals is essential.

The treatment responses varied within our cohort. Although most patients with CIDP respond well to IVIG, glucocorticoids, and plasma exchange therapy, patients with anti‐NF155‐antibody autoimmune nodopathy demonstrated less efficacy. Reports suggest that IVIG is effective in 13.1% of cases, glucocorticoids in 27.8%, and plasma exchange in 38.9% of cases, whereas rituximab is effective in 77.3% of cases.^28^ The lack of response to IVIG therapy in patients with anti‐NF155‐antibody autoimmune nodopathy could be attributed to the dominant isotype of the autoantibodies being IgG4. Indeed, IgG4 cannot activate the complement due to its inability to bind the first complement cascade component C1q and displays little FcR binding. The immunosuppressive effect of immunoglobulin through inhibition of the complement pathway has not been fully realized; therefore, the therapeutic effect remains poor.^29^ In our study, mRS scores were employed to assess drug efficacy, which indicated that IVIG monotherapy was ineffective, whereas the combination of rituximab and plasma exchange was effective. Rituximab is effective in managing patients early in the course of the disease and can improve patient prognosis. Patients with severe conditions often require a long recovery time and may need extended treatment. At the final follow‐up, all patients experienced symptom relief and had ceased glucocorticoid therapy, with four patients (patients 1, 2, 4, and 5) still undergoing rituximab treatment. However, the current study has certain limitations, including a limited sample size, lack of regular follow‐up, and the absence of post‐treatment monitoring for anti‐NF155 antibodies.

Previous studies have demonstrated that anti‐NF155 antibodies were present in 1%–23% of patients with CIDP.^13^, ^14^, ^15^, ^16^, ^30^, ^31^, ^32^ This variation in detection rates is probably due to a lack of uniformity in the diagnostic procedures. Although anti‐NF155 antibody testing is crucial for diagnosis, the current screening methods have limitations. Different detection methods, including enzyme‐linked immunosorbent assays, cell‐based flow cytometry, and western blotting, showed considerable variability in positive detection rates. These findings underscore the need for standardized testing protocols to enhance the reliability and reproducibility of anti‐NF155 antibody detection in clinical practice.

In summary, we recommend that patients with CIDP who present with specific clinical characteristics should be considered for anti‐NF155 antibody testing. Clinically, these patients often present with distal weakness, ataxia, hyperalgesia, limb numbness, and visual impairment. Significantly elevated CSF protein levels should be investigated further. Electrophysiological studies should demonstrate both motor and sensory dysfunctions. Furthermore, nerve ultrasound and spinal cord MRI should reveal nerve root thickening. Finally, a poor response to initial treatment with glucocorticoids and IVIG should be recognized as a critical factor that warrants consideration of alternative therapeutic approaches. For patients meeting these criteria, we propose early initiation of second‐line treatments, including rituximab and plasma exchange. Early diagnosis and intervention are paramount for mitigating the progression of disability and enhancing the overall prognosis of pediatric patients with CIDP. Timely detection of these antibodies can guide targeted and effective treatment strategies, potentially improving patient outcomes and quality of life.

## CONFLICT OF INTEREST

The authors declare no conflict of interest.
